# Mesoscale Simulation to Study Constitutive Properties of TATB/F_2314_ PBX

**DOI:** 10.3390/ma12223767

**Published:** 2019-11-16

**Authors:** Siyu Ge, Wenying Zhang, Jian Sang, Shuai Yuan, Glenn V. Lo, Yusheng Dou

**Affiliations:** 1 Chongqing Key Laboratory of Big Data for Bio Intelligence, Chongqing University of Posts and Telecommunications, Chongqing 40065, China; gesiyu96@163.com (S.G.); zhangwenying@cqupt.edu.cn (W.Z.); S170501007@stu.cqupt.edu.cn (J.S.); 2 Department of Chemistry and Physical Sciences, Nicholls State University, P.O. Box 2022, Thibodaux, LA 70310, USA; glenn.lo@nicholls.edu

**Keywords:** polymer bonded explosive (PBX), mesoscale, mechanical properties, constitutive relation, material point method

## Abstract

Material Point Method (MPM) mesoscale simulation was used to study the constitutive relation of a polymer bonded explosive (PBX) consisting of 1,3,5-triamino-2,4,6-trinitrobenzene (TATB) and a fluorine polymer binder F_2314_. The stress-strain variations of the PBX were calculated for different temperatures and different porosities, and the results were found to be consistent with experimental observations. The stress-strain relations at different temperatures were used to develop the constitutive equation of the PBX by using numerical data fitting. Stress-strain data for different porosities were used to establish the constitutive equation by fitting the simulation data to an improved Hashion-Shtrikman model. The equation can be used to predict the shear modulus and bulk modulus of the PBX at different densities of the sample. The constitutive equations developed for TATB/F_2314_ PBX by MPM mesoscale simulation are important equations for the numerical simulations of the PBX at macroscale. The method presented in this study provides an alternative approach for studying the constitutive relations of PBX.

## 1. Introduction

The development of constitutive models for polymer bonded explosives (PBXs), especially for load-deformation or stress-strain relationships, has received considerable attention in the energetic material field. The development of the constitutive equations usually starts with a series of experimental measurements with sophisticated equipment and techniques, followed by curve fitting of the experimental data based on a proposed model, and further testing and refinement by predicting mechanical properties [1,2,3,4].

This approach has two shortcomings. First, the design of experimental set up can be challenging, considering the uncertain and extremely sensitive behavior of explosives. Experiments are very expensive and difficult to carry out. Second, the constitutive models obtained in such a way are macroscopic approximate constitutive models [5,6], or empirical constitutive models [7,8]. These models fail to account for complex, multiphase, multiscale structural, dynamical, and chemical properties. For instance, the macroscale deformations applied to PBX composites in experiments are generally not the same as the local deformation fields in a component crystal within the composite. It is important for constitutive modeling to bridge the particulate nature at the mesoscale to the mechanical properties at the macroscale.

Computer simulation provides a safer and convenient way to study the mechanical properties of energetic material and has been used to assess and understand the constitutive relations of these materials. Most studies have been performed to understand the constitutive relations of energetic crystal materials or binders at atomic scale using molecular dynamics computation [9,10,11,12]. Only a few studies dealt with the mechanical properties of PBX composition at the microstate level.

The Material Point Method (MPM) [13,14,15] offers some advantages over other numerical techniques for simulations of mechanical properties of composites with complicated geometries found in PBX. It is easy in MPM to discretize complex geometries of PBX composites compared to mesh generation needed for Finite Element Method (FEM) calculations. Investigations reported by Banerjee et al. show that for simulations of large deformation of PBX with a high-strain rate, MPM has an advantage over other numerical methods [16,17,18].

This paper reports the mesoscale study of the constitutive relations of TATB/F_2314_ PBX using the MPM approach. We start with the constitutive equations and equations of state of both components, TATB grain, and F_2314_ binder and take the grain-grain, binder-binder, and grain-binder interactions into consideration. Based on the simulations results, the constitutive equations of TATB/F_2314_ PBX are developed and can be used in macroscale simulations. This study provides a numerical simulation approach to study the constitutive relation of PBX.

## 2. Methods and Simulation Model 

### 2.1. Material Point Method

MPM was described by Sulsky et al. [13]. The use of a regular background grid in MPM has a lot of computational advantages: it does not require periodic remeshing steps and remapping of state variables, and is, therefore, more suitable for problems involving contact between materials, cracks, large deformations, and other high-velocity problems [14]. 

In MPM, materials are discretized into particles on a background grid and each particle is assigned a mass, coordinate, velocity, and other properties. The discrete momentum equation is given as:

(1) $$ma=f^{ext}-f^{\mathrm{int}}$$

where m is the mass matrix, a is the acceleration vector, $f^{ext}$ is the external force vector, and $f^{\mathrm{int}}$ is the internal force vector resulting from the divergence of the material stresses.

In each MPM step, all particle states are extrapolated to the nodes of the computational grid, then the mass matrix m, the nodal external forces $f^{ext}$, and velocities v are calculated at individual nodes using the following equations:

(2) $$m_{i}=\sum_{p}S_{ip}m_{p}$$

(3) $$v_{i}=(1/m_{i})\sum_{p}S_{ip}m_{p}v_{p}$$

(4) $$f_{i}^{ext}=\sum_{p}S_{ip}f_{p}^{ext}$$

where ∑ represents a summation over all particles, i refers to individual nodes of the grid. $m_{p}$ is the particle mass, $v_{p}$ is the particle velocity, $f_{p}^{ext}$ is the external force on the particle, and $S_{ip}$ is the generalized shape function of the node i evaluated at the particle p.

The nodal internal forces $f_{i}^{\mathrm{int}}$ is computed at the nodes as a volume integral of the divergence of the stress on the particles:

(5) $$f_{i}^{\mathrm{int}}=\sum_{p}G_{ip}\sigma_{p}V_{p}$$

where $G_{ip}$ is the shape function gradients of the node i evaluated at the particle p, while $\sigma_{p}$ and $V_{p}$ are stress and volume over the particle domain, respectively.

As the momentum equations for grid nodes are solved, then mapped back to the material points, and state variables are calculated at the material points using the updated kinematic variables.

### 2.2. Interface friction Contact Algorithm

PBX consists of TATB grains and F_2314_ polymeric binder, while F_2314_ is a fluoropolymer binder containing a 1:4 molar ratios of comonomer vinylidene fluoride (VDF) and chlorotrifluoroethylene (CTFE). F_2314_ is one of the most commonly used polymer binders for PBX [19]. Two components of PBX have different constitutive models and therefore, interface interaction between two components should be taken into consideration in mesoscale simulation. The interaction of material interfaces can be realized using the Multi-material algorithm [20]. In Multi-material MPM, each material extrapolates to its own velocity field. All nodes with more than one velocity field must determine if the materials are in contact. When the surfaces are determined to be in contact, the task is to modify nodal momenta and forces to reflect the implemented contact mechanics; details of the algorithm are as follows:

To implement friction between TATB and F_2314_, the tangential traction experienced by frictional sliding is calculated by:

*S_slide_* = *g*(*N*, *A_c_*, Δ**v**′*_i_*, …) (6)

where $S_{slide}$ is sliding traction and $g\left(N,A_{c},\Delta {v^{\prime }}_{i},....\right)$ is any arbitrary friction law which may depend on various parameters such as contact normal pressure N, contact area $A_{c}$, and relative sliding velocity $\Delta {v^{\prime }}_{i}$. 

An improved multi-material method was used to model contact between TATB and binder and the sliding force was calculated by:

(7) $$S_{slide}A_{c}\Delta t=S_{a}A_{c}\Delta t-\mu d_{n}$$

where $S_{a}$ is a shear adhesion strength of the interface, μ is the coefficient of friction, and $d_{n}$ is the normal momentum changes required to stick. $S_{a}$ takes zero in this calculation because for surfaces adhesion, the calculation is equivalent to MPM treatment of self-contact [20]. The calibration of interfacial parameters is taken from the literature where available.

### 2.3. Simulation Model

In this mesoscale interface model for TATB-based PBX, the analysis focuses on a two-phase meso-structure consisting of TATB grains and F_2314_ binder. In MPM code [21], each material has a different constitutive equation and equation of state and two materials are discretized into material points. They carry the history-dependent state variables such as stresses and strains, as well as mass and kinematic variables such as position, velocity, and acceleration. An interface between materials is considered and all those state variables for PBX then are calculated at the material points using the updated kinematic variables of the two materials and their interfaces.

An elastic-plastic model and Mie-Grüneisen equation of state are used for TATB grains. The elastic-plastic material model [22] for deviatoric stress is expressed as:

(8) $$\sigma =[1+{\left(\dot{\varepsilon }/C_{c}\right)}^{\frac{1}{P_{c}}}](\sigma_{0}+\beta E_{P}\varepsilon_{P}{}^{eff})$$

where, σ is stress, $\sigma_{0}$ is initial yield stress, all units are MPa, $\dot{\varepsilon }$ is the strain rate, and $C_{c}$ and $P_{c}$ are Cowper-Symond strain rate parameters. $\varepsilon_{P}{}^{eff}$ is the effective plastic strain, $E_{P}$ is plastic hardening modulus, and β is the hardening parameter. The Cowper Symonds strain rate parameters were sent to zero as a constant strain rate was applied and only one yielded stress for PBX.

The Mie-Grüneisen equation [22] of state is given by:

(9) $$P=\frac{\rho c^{2}\mu [1+(1-\gamma_{0}/2)\mu -\alpha \mu^{2}/2]}{\left[1-(s_{1}-1)\mu -s_{2}\mu^{2}/(\mu +1)-s_{3}\mu^{3}/(\mu +1)\right]}+(\gamma_{0}+\alpha \mu )E$$

where, P is pressure; $\mu =\rho /\rho_{0}-1=1-V_{0}/V$, $\rho_{0}$ is for initial density, $V_{0}$ is initial volume; c is the bulk speed of sound at room temperature and pressure; $\gamma_{0}$ is Grüneisen’s gamma at the reference state; E is internal energy per unit reference volume; $s_{1}$,$s_{2}$, and $s_{3}$ are linear Hugoniot slope coefficients, and α is a first order volume correction factor.

A viscoelastic constitutive model [23] and Tait equation [24] of state are used for the F_2314_ binder. The Cauchy stress for F_2314_ binder is expressed as:

(10) $$\sigma =\int_{0}^{t}2G(t-\tau )\frac{de}{d\tau }d\tau +I\int_{0}^{t}K(t-\tau )\frac{d\Delta }{d\tau }d\tau$$

where σ represents the Cauchy stress. G(t) and K(t) are the deviatoric part of the shear relaxation modulus and bulk modulus of the constitutive behavior, respectively. e and Δ refer to the deviatoric and hydrostatic portions of the Eulerian strain tensor, and t and τ refer to physical and reduced times, respectively. I is the unit tensor.

The Tait equation of state is expressed as:

(11) $$\frac{V\left(T,P\right)}{V_{0}\left(T\right)}=1-C\ln\left(1+\frac{P}{B(T)}\right)$$

where V(T,P) represents the final volume of compression at temperature T and pressure P; $V_{0}\left(T\right)$ is the temperature dependence of the volume at zero pressure. The parameter B is a function of temperature and depends on the specific system, and B(T)=CK(0,T). The parameter C is independent of structure and temperature, while K(0,T) is the temperature dependence of the bulk modulus at zero pressure.

The parameters of TATB grain [25,26] and F_2314_ [27] binder used in this study are listed in Table 1.

In this study, an idealized meso-structure, with packed circular particles, was chosen for TATB grains. Research reported by Barua et al [28] showed that the particle morphology of explosive particles has some effects on the interface interaction, interface debonding, and energy distribution, while it has little effect on the effective elastic response of PBX, as reported by Dai [29] et al. 

**Figure 1:**
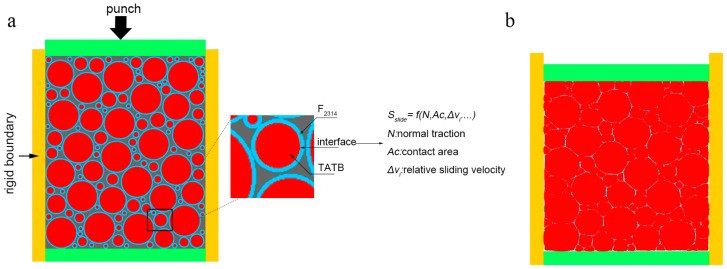
(**a**) Uniaxial compression model of PBX on mesoscale, (**b**) snapshot of final state of compression.

All particles are placed randomly within the packing box with a bimodal distribution of the diameters, which is consistent with experimental observations [30]. The mesoscopic calculation model based on the MPM was designed as shown in Figure 1a. The model consists of a rigid mold, rigid punch, TATB grains, F_2314_ binder, and void between particles. Furthermore, interfacial properties between particles and binders that describe frictional contact were included in our model. The calculations were performed on a rectangular region of 1.0 × 1.2 mm. Materials are confined between three fixed rigid walls and a fourth wall that moves at a constant velocity of 0.3 m/s, which is about 0.01% of the bulk wave speed for the TATB grains in PBX. The velocity of the top surface yields overall strain rates of 250 s^−1^. The diameters of TATB grains are chosen based on experimental data [30]. However, a minimum diameter is not selected, this results in a size range from 0.05 mm to 0.3 mm. F_2314_ binder thickness ranging from 0.0013 to 0.0078 mm was selected to cover the outer layer of particles. The mass ratio between TATB and the binder was 95:5. The friction coefficient of 0.3 was used in the calculation. This value was also used for frictional contact between HMX particles and binder in an earlier simulation [20], where the HMX particles and binder were modeled with Mie-Grüneisen pressure response and an elastic shear response. The simulation results were consistent with experimental observation. Figure 1b shows the snapshot of final state of compression. The snapshot is similar to the realistic PBX morphology, and is used for discussions. 

A compression simulation was performed for different temperatures. Temperature-force coupled loading was performed on the simulation model of the same fill rate with an initial porosity of 0.08 and a corresponding density of 1.787 g/cm^3^. The four loading temperatures are 293 K, 333 K, 353 K, and 373 K. The initial temperature of the PBX particles and surrounding environment was set to the corresponding temperature. Simulations using different densities of the PBX sample (1.761 g/cm^3^, 1.787 g/cm^3^, and 1.839 g/cm^3^) with an initial temperature of 293 K, were performed to study the effect of porosity on mechanical properties as well.

## 3. Results and Discussion

### 3.1. Calculation of Mechanical Properties at Different Temperature

**Figure 2:**
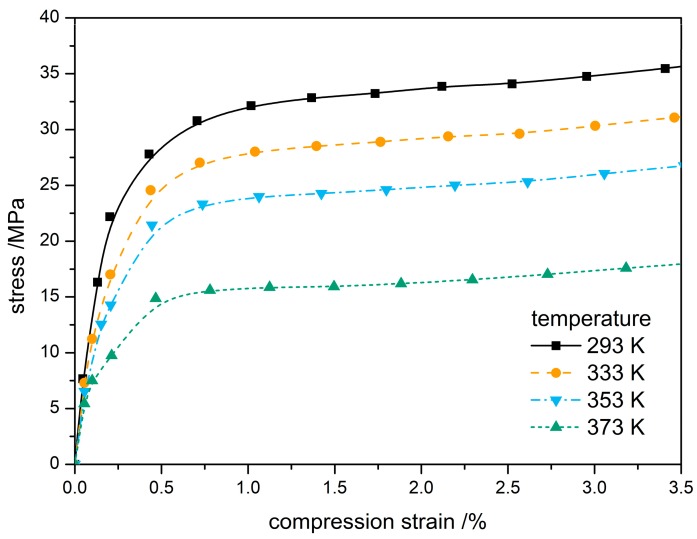
Stress-strain curve of PBX at different temperatures.

Stress and strain plots for compression of the PBX are presented in Figure 2 for the temperatures at 293 K, 333 K, 353 K, and 373 K. The stress-strain variations with different temperatures all show two stages: a fast-linear rise at early time followed by a slow non-linear variation, due to the elastic behavior and plastic deformation behavior of the PBX, respectively. Young’s modulus, the slope of the linear changes of stress in the linear stage, clearly decreases with increasing temperatures. When the linear stage terminates at what is known as the yield point, stress changes slowly with strain. The yield stress of PBX estimated from the curve levels of stress-strain variations and the beginning of plastic deformation ranges from 13 MPa to 25 MPa and decreases as temperature increases. The yield stress of PBX is obviously lower than that of TATB, indicating the influence of binder on the properties and response of TATB. The notable temperature dependence of quasi-static compression for PBX comes from the fact that polymeric binder softening at high temperature affects the interface interaction between binders and particles and, therefore, changes the mechanical properties of PBX. The simulation results demonstrate the value of Young’s modulus and yield stress decrease as the loading temperature increases, indicating a negative correlation of temperature with mechanical properties. However, the critical strains of PBX transition from linear elasticity to elastic-plastic stage were not affected. All these results are consistent with the experimental results of Guo [31] et al.

**Figure 3:**
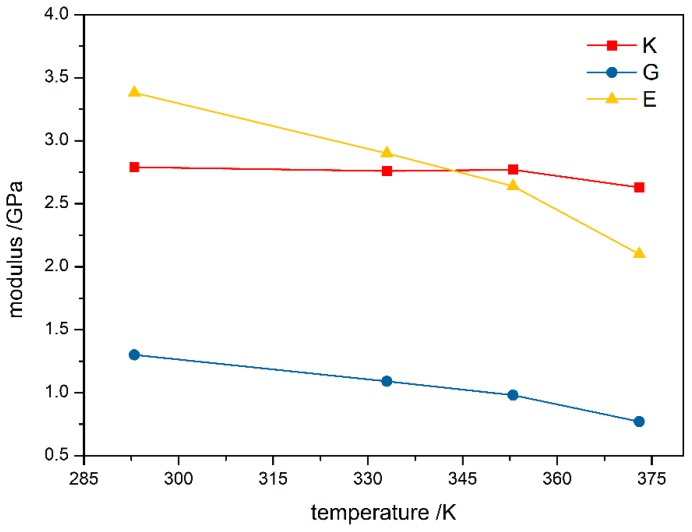
Modulus curve of PBX at different temperatures. K = bulk modulus, G = shear modulus, and E = Young’s modulus.

The change of bulk modulus, shear modulus, and Young’s modulus with temperatures are plotted in Figure 3. The elastic modulus of PBX was negatively correlated with temperature change. Bulk modulus did not change significantly from 293 K to 353 K because of the property of F_2314_ binder, meaning the binder softening due to temperature can be restored when the loading temperature is not particularly high. The three elastic moduli for TATB-based PBX at different temperatures obtained by fitting the stress-strain curves are compared with values from experiment [32] and molecular dynamics simulation [33] in Table 2. All the moduli decrease with increasing loading temperature because the flexibility of polymeric binder chain segments increases at higher temperature. This leads to an increase in the elasticity of PBX. The bulk modulus and shear modulus for PBX at 293 K are close to those obtained from the molecular dynamic simulation [33], and Young’s modulus at 298 K is close to experimental observation [32]. In addition, Young’s modulus at 333 K is in consistent with experimental values from 323 to 338 K [32].

**Figure 4:**
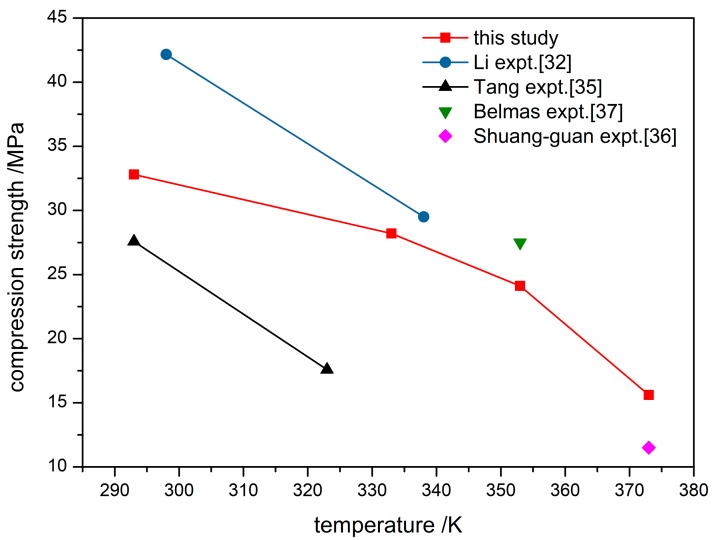
Compression strength curve of PBX at different temperatures, also shown are experimental results from references [32,35,36,37].

The compression strength of PBX at different temperatures obtained from stress-strain variations is plotted in Figure 4. The compression strength decreases with temperature and a rapid decrease above 353 K results from binder softening. Experimental dynamic mechanical thermal analysis of F_2314_ [34] have shown that the behavior of F_2314_ binder with temperature falls into following steps: glassy state, the transition zone between glassy state and rubber state, and rubber state, followed by the change to the viscous flow state. In the viscous flow state, the binder loses more adhesion to explosive particles and the compression strength of PBX decreased significantly. Experimental results [32,35,36,37] of PBX compression strength are shown in Figure 4 for comparison. Different experimental data come from either different techniques or different temperatures. The simulation results of this study are within the range of these data.

The aforementioned comparisons show that the results of MPM mesoscale simulation are reliable and can be applied to develop the constitutive model for the mechanical properties of TATB/F_2314_ PBX. Based on the stress-strain variations from MPM mesoscale simulation, the constitutive equation of the PBX can be established in the following steps [38].

**Figure 5:**
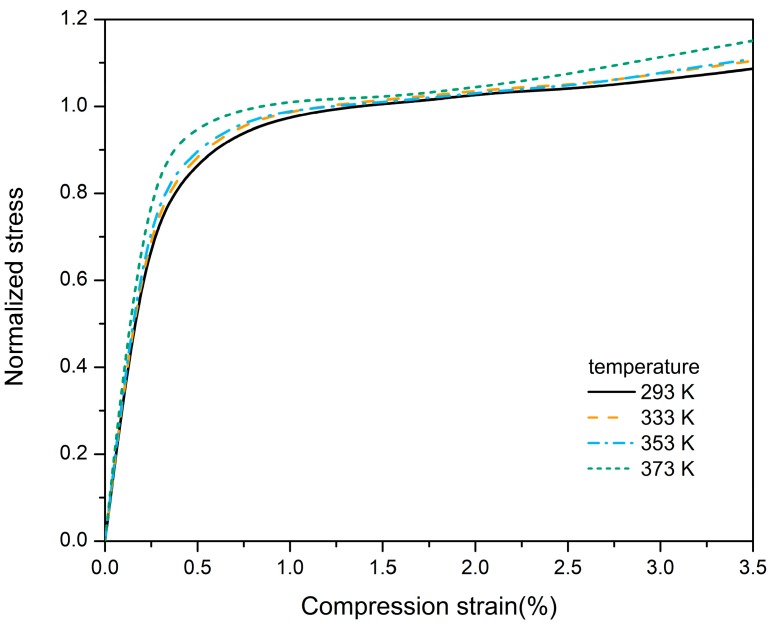
Stress-strain curves of PBX after being stress normalized.

First, normalized stress $\sigma_{n}$ can be obtained as follows:

(12) $$\sigma_{n}=\sigma /\sigma_{c}$$

where $\sigma_{c}$ and σ are the compression strength and compression stress respectively. The stress-strain curves of PBX at different temperatures are normalized and plotted in Figure 5. The obtained stress-strain curves at different temperatures are basically coincident and therefore the stress-strain curve fitting can be performed at a fixed temperature.

The relationship between compression strength and loading temperature is expressed as:

(13) $$\sigma_{c}=\sigma_{0}\cdot \exp(\alpha (1-\frac{T}{T_{0}}))$$

where $T_{0}$ is reference temperature and takes a value of 293 K, $\sigma_{0}$ is the compression strength at reference temperature, and α refers to a temperature effect factor. Each temperature has a corresponding compression strength that is listed in Table 3. $\sigma_{0}$ and α can be obtained by fitting the data in Table 3 into equation 13; the results are: $\sigma_{0}$ = 34.10 MPa and α = 2.357.

The stress-strain relationship can be obtained by fitting the normalized stress-strain curve as follows:

(14) $$\sigma_{n}=A\varepsilon +B\varepsilon^{n}$$

where the first term refers to the elastic stage, and the second term refers to visco-plastic stage. Parameters A, B, and n can be approximately regarded reflecting the essential properties of material and are independent of loading temperature. Normalized stress and strain data at 293 K in Figure 5 are used in the fitting process and the parameters were found to be: A = −0.1206, B = 1.084, and n = 0.2467.

The constitutive equation eventually obtained by combining Equations (12)–(14) is:

(15) $$\sigma =[\sigma_{0}\cdot \exp(\alpha (1-\frac{T}{T_{0}}))]\cdot (A\varepsilon +B\varepsilon^{n}).$$

**Figure 6:**
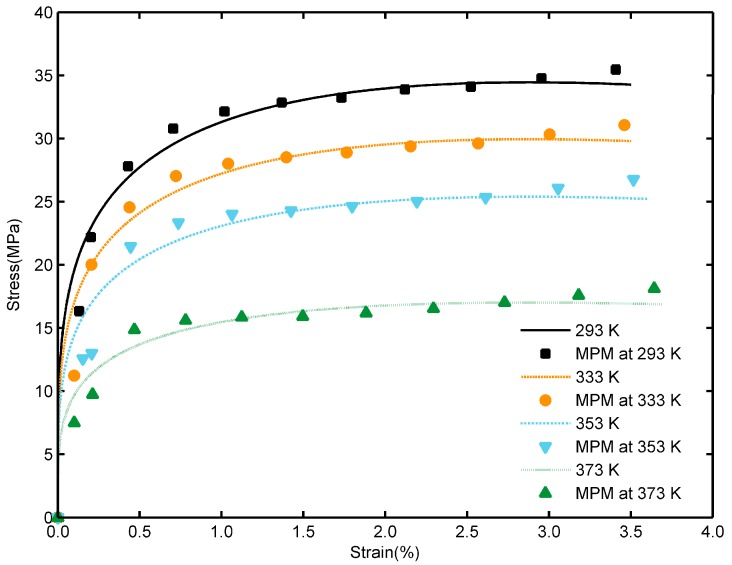
Constitutive curves fitted at different temperatures; data points are simulation results of MPM.

Figure 6 shows the variations of the stress of TATB/F_2314_ PBX against strain calculated from the constitutive equation at the different temperature. The simulation results are also plotted for comparison. The stress-strain curve shows two stages: linear elastic deformation and a long plateau region after yielding. The plots calculated by the constitutive equation are in good agreement with simulation results. Experimental study [39] investigated the local damage initiation mechanism of polymer bonded sugar (PBS) and found that the stress-strain curve of PBS shows the similar behaviors. However, the experimentally found failure strain of PBS at about 6.5% was not observed in this study due to the absence of local failure strain for small strain deformation. The constitutive equations developed for TATB/F_2314_ PBX for a mesoscale simulation can be a bridge for the numerical simulations at macroscale.

### 3.2. Mechanical Properties with Different Porosity

**Figure 7:**
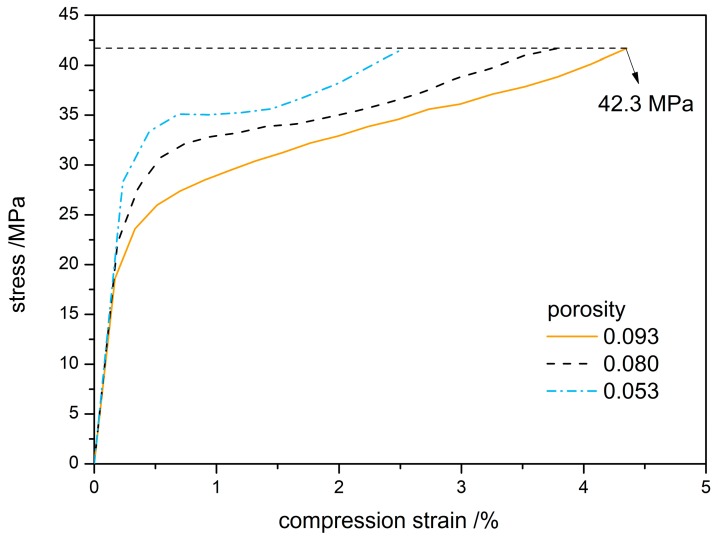
Stress-strain curve of PBX at different porosity levels.

Figure 7 gives stress-strain curves of TATB-based PBX at different porosities. The stress-strain dependence on porosity follows the same trends. When the compression strain is smaller than 0.5%, the slope of the stress-strain curve increases with the increase of density, indicating that the elastic modulus of the PBX increases as the porosity of the sample decreases in the linear elastic stage. As the strain goes above 0.5%, the compression enters the plastic yield stage, where the stress varies slowly with the strain. At 99.0% theoretical density, all three porosity samples reach the stress value of 42.3 MPa. This result is supported by the experimental observation [40] that the pores between explosive particles almost disappeared when the loading force reached 40 MPa during the compaction of TATB granules.

**Figure 8:**
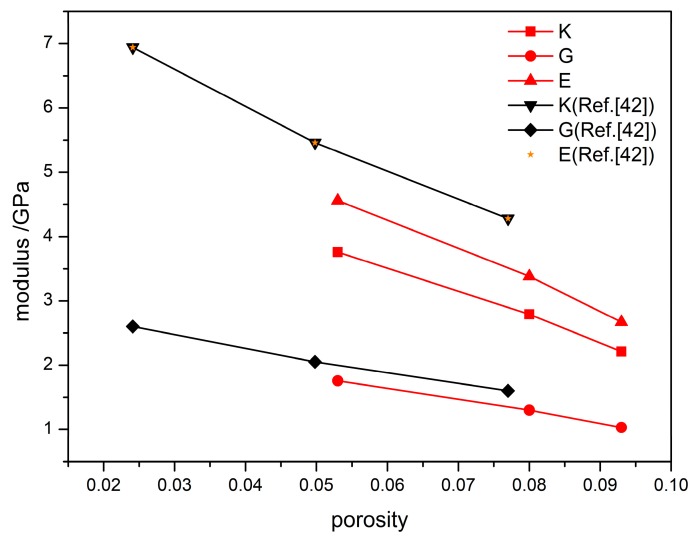
Modulus curve of PBX at different porosities, as well as experimental data from reference [41], K = bulk modulus, G = shear modulus, and E = Young’s modulus.

Figure 8 presents the variations of bulk modulus, shear modulus, and Young’s modulus for different porosities. All three moduli increase as the porosity decreases and this is consistent with experimental observations [41]. This study suggests that the smaller porosity of initial filling leads to higher compression strength and elastic modulus of PBX and, therefore, to better mechanical properties. Research found [42] that the initial damage is associated with pores in a compressed device and a poor density uniformity always appears in the parts where pores exist. These parts may become the failure zones owing to the existence of microcracks. Figure 8 shows experimental data for comparison. It is seen that the variation trend of PBX modulus with porosity obtained in this study is in good agreement with that of experimental results and that the simulated values are consistent with experimental values.

The stress-strain variations and modulus changes presented in Figure 7 and Figure 8 reveal that the mechanical properties of PBX depend on the porosity systemically and that the compression strength and elastic modulus both significantly depend on the porosity of initial filling. Based on these factors, an improved Hashion-Shtrikman model [41] was used to fit the simulation data for the mechanical properties of different porosity.

For the improved Hashion-Shtrikman model, shear modulus and bulk modulus can be expressed as:

(16) $$G_{eff}=f\left(\rho /\rho_{0}\right)G^{U}$$

(17) $$K_{eff}=f\left(\rho /\rho_{0}\right)K^{U}$$

where $f\left(\rho /\rho_{0}\right)$ refers to interface bonding parameter, $\rho_{0}$ is the theoretical maximum density, ρ is the actual density of PBX, the theoretical calculation values, and $G^{U}$ and $K^{U}$ are upper bounds for effective shear modulus and effective bulk modulus in Hashion-Shtrikman model, respectively. These parameters take the values from reference [41]: $G^{U}$ = 10.84 GPa, and $K^{U}$ = 10.52 GPa.

The actual density is expressed as $\rho =\frac{1-\phi }{v}$, where ϕ refers to porosity and v is the specific volume of material: $v=\frac{\sigma_{p}}{\rho_{p}}+\frac{\sigma_{b}}{\rho_{b}}$, of which $\sigma_{p}$, $\rho_{p}$, $\sigma_{b}$, $\rho_{b}$ refer to mass percentage and the density of the particle and binder, respectively.

Considering the correlation between theoretical density and actual density, $f(\rho /\rho_{0})$ can be set as:

(18) $$f(\rho /\rho_{0})=k{\left(\rho /\rho_{0}\right)}^{n}$$

where k and n are parameters related to each effective modulus.

Three porosities of PBX (0.0932, 0.0798, and 0.0530) were used for this study. When porosity reaches 0, the theoretical maximum density of PBX is 1.942 g/cm^3^. Equations (19) and (20) can be obtained from the improved Hashion-Shtrikman Equations (16) and (17):

(19) $$\ln k+n\ln\left(\rho /\rho_{0}\right)=\ln\left(G_{eff}/G^{U}\right)$$

(20) $$\ln k+n\ln\left(\rho /\rho_{0}\right)=\ln\left(K_{eff}/K^{U}\right)$$

where $G_{eff}$ and $K_{eff}$ are the values from this simulation study and k and n can be obtained from linear fitting of the simulation data. The resulting constitutive equations for shear modulus and bulk modulus with respect to porosity are:

(21) $$\left(G/G^{U}\right)=0.318{\left(\rho /\rho_{0}\right)}^{12.11}$$

(22) $$\left(K/K^{U}\right)=0.696{\left(\rho /\rho_{0}\right)}^{12.01}.$$

**Figure 9:**
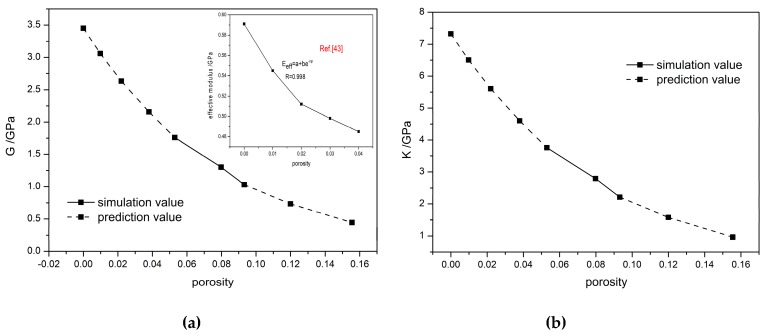
Prediction results of (**a**) shear modulus and (**b**) bulk modulus of PBX, also shown in (**a**) as the simulation results curve from reference [43].

Equations (21) and (22) can be used to predict the modulus of TATB/F_2314_ PBX with different densities. The predicted variations of two moduli at different porosities are plotted in Figure 9a,b, respectively. The figures show that the shear modulus and bulk modulus of the PBX decreased exponentially with the density. A finite element simulation reported by Wei et al. [43] found that the effective elastic modulus of TATB-based PBX decreases exponentially with porosity, and these results were also shown in Figure 9a. It is worth noting that a discrepancy of predictive shear modulus occurs at lower porosity compared to finite element simulation results [43]. The discrepancy comes from the differences in the loading conditions and systems simulated: In the finite element simulation, TATB and binder have a mass ratio of 50:50. However, in the present calculation, the mass ratio between TATB and binder is 95:5. Increasing proportion of binder results in a significant decreasing of the effective elastic modulus. Moreover, the loading rates are completely different in two studies, which also cause a discrepancy of the shear modulus. However, the predictive shear modulus at low porosity is close to simulation result at 293 K [33], which indicates that the tendency of predictions is reliable.

## 4. Conclusions

Starting with the constitutive equations and the equations of state for both TATB grain and F2314 binder, the mechanical properties obtained from MPM mesoscale simulation can be applied to derive the constitutive relations of TATB/F_2314_ PBX. The constitutive equations of the PBX obtained from MPM simulation can be used to numerically determine mechanical properties of the PBX at a macroscale.

The stress-strain variations of TATB/F_2314_ PBX obtained from MPM mesoscale simulation for different temperatures were consistent with available experimental observations. The simulation results can be fitted numerically to the well-known constitutive law to determine the parameters usually obtained from sophisticated experiments for the development of constitutive equations.

The stress-strain changes of the PBX for different porosities computed by MPM mesoscale simulation are in reasonable agreement with available experimental observations or other numerical simulations. The computed properties can be fitted to an improved Hashion-Shtrikman equation to determine the parameters that are important to the constitutive equations of the PBX which can be used to predict the shear modulus and bulk modulus of the PBX at different densities (and, therefore, different porosities).

The development of the constitutive relationship is an important research topic in the field and the method presented in this paper provides a numerical approach for the investigation of the constitutive equations. This would be useful for multiple scale simulation of PBX, where the computation is carried from microscale to macroscale.

## Figures and Tables

**Table 1:** The model parameters of Trinitro-1,3,5-benzenetriamine (TATB) ^1^ and F_2314_^2^.

Material	Parameters	Values
TATB	shear modulus (GPa)	11.5
	density (g/cm^3^)	1.934
	yield stress (GPa)	0.80
	$C_{c}$	0
	$P_{c}$	0
	c (m/s)	2340
	Grüneisen $\gamma_{0}$	1.60
	$s_{1}$	2.32
	$s_{2}$	0
	$s_{3}$	0
F_2314_	bulk modulus (GPa)	0.33
	shear modulus (GPa)	0.07
	density (g/cm^3^)	2.02
	C	0.08936
	K(0,T) (GPa)	0.27

^1^ Parameter values of TATB were taken from references [25,26]; ^2^ Parameter values of F_2314_ were taken from reference [27].

**Table 2:** Mechanical properties of PBX at different temperatures. K = bulk modulus, G = shear modulus, and E = Young’s modulus.

Temperature (K)	K (GPa)	G (GPa)	E (GPa)
293 ^1^	2.79	1.30	3.38
293 ^2^	2.22	2.21	4.98
298 ^3^	–	–	3.20
323 ^3^	–	–	2.92
333 ^1^	2.76	1.09	2.90
338 ^3^	–	–	2.48
353 ^1^	2.77	0.98	2.64
373 ^1^	2.63	0.77	2.10

^1^ Simulation results of this study; ^2^ simulation results in [33]; ^3^ experimental results in [32].

**Table 3:** Compression strength of PBX at different temperatures.

Temperature (K)	Simulation Compression Strength (MPa)
293	32.8
333	28.2
353	24.1
373	15.6

## References

[1] Skidmore C.B., Idar D.J., Buntain G.A., Son S.F., Sander R.K. Aging and PBX 9502. Proceedings of the Los Alamos National Laboratory Report LA-UR-98-1206, Conference on Life Cycles of Energetic Materials.

[2] Groves S., Cunningham B. Tensile and Compressive Mechanical Properties of Billet Pressed LX17-1 as a Function of Temperature and Strain Rate. Proceedings of the Lawrence Livermore National Laboratory Report UCRL-ID-137477, Office of Scientific & Technical Information Report.

[3] Govier R.K., Iii G.T.G., Blumenthal W.R. (2008). Comparison of the Influence of Temperature on the High-Strain-Rate Mechanical Responses of PBX 9501 and EDC37. Metall. Mater. Trans. A.

[4] Lin Y., Xu B., Chen R., Qin J., Lu F. (2014). Dynamic Mechanical Properties and Constitutive Relation of an Aluminized Polymer Bonded Explosive at Low Temperatures. Shock Vib..

[5] Guo H., Luo J.R., Shi P.A., Xu J.G. (2014). Research on the fracture behavior of PBX under static tension. Def. Tech..

[6] Xiao Y., Yi S., Zhen Y., Guo L., Yao L. (2017). Characterization, modeling and simulation of the impact damage for polymer bonded explosives. Int. J. Impact Eng..

[7] Bischoff P.H., Perry S.H. (1991). Compressive behavior of concrete at high strain rates. Mater. Struct..

[8] Bennettb J.G., Haberman K.S., Johnson J.N., Asay B.W., Henson B.F. (1998). A constitutive model for the non-shock ignition and mechanical response of high explosives. J. Mech. Phys. Solids.

[9] Smith G.D., Menikoff R. (2003). Molecular dynamics simulation study of elastic properties of HMX. J. Chem. Phys..

[10] Dmitry B., Oleg B., Smith G.D., Sewell T.D., Dattelbaum D.M., Stevens L.L. (2009). A molecular dynamics simulation study of crystalline 1,3,5-triamino-2,4,6-trinitrobenzene as a function of pressure and temperature. J. Chem. Phys..

[11] Byrd E.F.C., Rice B.M. (2007). Ab Initio Study of Compressed 1,3,5,7-Tetranitro-1,3,5,7-tetraazacyclooctane (HMX), Cyclotrimethylenetrinitramine (RDX), 2,4,6,8,10,12-Hexanitrohexaazaisowurzitane (CL-20), 2,4,6-Trinitro-1,3,5-benzenetriamine (TATB), and Pentaerythritol Tetranitrate (PETN). J. Phys. Chem. C.

[12] Vu-Bac N., Areias P.M.A., Rabczuk T. (2016). A multiscale multisurface constitutive model for the thermo-plastic behavior of polyethylene. Polymer.

[13] Sulsky D., Chen Z., Schreyer H.L. (1993). A particle method for history-dependent materials. Comput. Methods Appl. Mech..

[14] Sulsky D., Zhou S.J., Schreyer H.L. (1995). Application of a particle-in-cell method to solid mechanics. Comput. Phys. Commun..

[15] Sulsky D., Schreyer H.L. (1996). Axisymmetric form of the material point method with applications to upsetting and Taylor impact problems. Comput. Methods Appl. M.

[16] Banerjee B., Guilkey J.E., Harman T.B., Schmidt J.A., Mcmurtry P.A. Simulation of impact and fragmentation with the material point method. Proceedings of the 11th International Conference on Fracture.

[17] Banerjee B. (2004). Validation of the Material Point Method and Plasticity with Taylor Impact Tests.

[18] Banerjee B. Material point method simulations of fragmenting cylinders. Proceedings of the 17th ASCE Engineering Mechanics Conference.

[19] Sun J., Dong H.S., Zheng P.S., Zhang L. (2000). Study on Fluoropolymer F_2314_ Bonded HMX. Chin. J. Energy Mater..

[20] Nairn J.A., Bardenhagen S.G., Smith G.D. (2017). Generalized contact and improved frictional heating in the material point method. Comput. Part. Mech..

[21] NarinMPM Simulation Package Is Freely. Each Element Contained 4 Material Points. Update Strain Average Option Was Used. http://nairn.mse.utah.edu/.

[22] Wang C., Chen L., Liu Q., Pi Z.D., Hu X.M. (2014). Numerical simulation for analyzing shock to ignition of PBXs with different compositions in meso-structural level. Explos. Shock Waves.

[23] Seidel G.D., Allen D.H., Helms K.L.E., Groves S.E. (2005). A model for predicting the evolution of damage in viscoelastic particle-reinforced composites. Mech. Mater..

[24] Davande H., Bedrov D., Smith G.D. (2008). Thermodynamic, Transport, and Viscoelastic Properties of PBX-9501 Binder: A Molecular Dynamics Simulations Study. J. Energ. Mater..

[25] Zhu W., Xiao J.J., Huang H., Ma X.F., Li J.S., Xiao H.M. (2007). Temperature effect on mechanical properties of TATB and TATB/F_2311_ PBX by molecular dynamics simulation. J. Nanjing Univ. Sci. Technol..

[26] Hobbs M.L., Baer M.R., Gross R.J. A constitutive mechanical model for energetic materials. Proceedings of the Sandia National Laboratory Report SAND-94-0363C, International Pyrotechnics Seminar.

[27] Jing S.M. (2009). Study on the Effective Mechanical Properties and Constitutive Relations of PBX. Master’s Thesis.

[28] Barua A., Horie Y., Zhou M. (2012). Energy localization in HMX-Estane polymer-bonded explosives during impact loading. J. Appl. Phys..

[29] Dai K.D., Liu Y.L., Chen P.W., Tian Y. (2012). Finite element simulation on effective elastic modulus of PBX explosives. Trans. Beijing Inst. Technol..

[30] Skidmore C.B., Phillips D.S., Son S.F., Asay B.W., Schmidt S.C., Dandekar D.P., Forbes J.W. (1998). Characterization of HMX particles in PBX 9501. Shock Compression of Condensed Matter-1997.

[31] Guo H.F., Wu Y.Q., Huang F.L. (2018). Wide range temperature-dependent deformation and fracture mechanisms for 8701 under dynamic and static loading. RSC Adv..

[32] Li J.L., Lu F.Y., Chen R., Qin J.G., Zhao P.D., Lan L.G., Jing S.M. (2011). Dynamic Behavior of Three PBXs with Different Temperatures. J. Dyn. Behav. Mater..

[33] Shu Y., Yi Y., Huo J., Liu N., Wang K., Lu Y., Wang X., Wu Z., Shu Y., Zhang S. (2017). Interactions between poly-(phthalazinone ether sulfone ketone) (PPESK) and TNT or TATB in polymer bonded explosives: A molecular dynamic simulation study. J. Mol. Model..

[34] Tang M.F., Wen M.P., Tu X.Z. (2018). Influence and Mechanism of High Temperature and Mechanical Stress on the Mechanical Behaviors of PBXs. Chin. J. Energy Mater..

[35] Tang W., Yuan H.W., Wen M.P. (2018). Quasi-static tension-compression nonlinear constitutive model of TATB-based PBX and its application. Chin. J. Energy Mater..

[36] Shang-Guan P.J. (2017). Digital Image Correlation Method on Studying Compressive Mechanical Properties of PBX in Wide Temperature Range. Master’s Thesis.

[37] Belmas R., Plotard J.P. (1995). Physical Origin of Hot Spots in Pressed Explosive Compositions. J. Phys. IV.

[38] Chen R., Fang-Yun L.U., Lin Y.L., Wang R.F. (2007). Mechanical Behavior and Constitutive Model of Pressed Aluminized Explosive. Chin. J. Energy Mater..

[39] Dai B. (2015). Study on Structure Evolution and Stress Response of Modeling Particles in TATB Molding Process. Master’s Thesis.

[40] Ravindran S., Tessema A., Kidane A. (2017). Multiscale damage evolution in polymer bonded sugar under dynamic loading. Mech. Mater..

[41] Jing S.M., Ming L.I., Long X.P. (2009). On Predicting Effective Elastic Modulus of PBX by Modified Hashin-Shtrikman Model. Chin. J. Energy Mater..

[42] Yang K., Wu Y., Huang F. (2018). Numerical simulations of microcrack-related damage and ignition behavior of mild-impacted polymer bonded explosives. J. Hazard. Mater..

[43] Wei X.W., Li M., Li J.-M., Tu X.-Z., Zhou M.L. (2014). Influence of Pore Properties on Effective Elastic Modulus of TATB Based Polymer Bonded Explosive. Chin. J. Energy Mater..

